# Hydroxylation Increases the Neurotoxic Potential of BDE-47 to Affect Exocytosis and Calcium Homeostasis in PC12 Cells

**DOI:** 10.1289/ehp.11059

**Published:** 2008-02-01

**Authors:** Milou M.L. Dingemans, Aart de Groot, Regina G.D.M. van Kleef, Åke Bergman, Martin van den Berg, Henk P.M. Vijverberg, Remco H.S. Westerink

**Affiliations:** 1 Toxicology Division, Institute for Risk Assessment Sciences, Utrecht University, Utrecht, the Netherlands; 2 Department of Environmental Chemistry, Wallenberg Laboratory, Stockholm University, Stockholm, Sweden

**Keywords:** bioactivation, brominated flame retardants, calcium, catecholamine, exocytosis, intra-cellular calcium stores, neurotoxicity, neurotransmitter release, persistent organic pollutants, poly-brominated diphenyl ether

## Abstract

**Background:**

Oxidative metabolism, resulting in the formation of hydroxylated polybrominated diphenyl ether (PBDE) metabolites, may enhance the neurotoxic potential of brominated flame retardants.

**Objective:**

Our objective was to investigate the effects of a hydroxylated metabolite of 2,2′,4,4′-tetra-bromodiphenyl ether (BDE-47; 6-OH-BDE-47) on changes in the intracellular Ca^2+^ concentration ([Ca^2+^]*_i_*) and vesicular catecholamine release in PC12 cells.

**Methods:**

We measured vesicular catecholamine release and [Ca^2+^]*_i_* using amperometry and imaging of the fluorescent Ca^2+^-sensitive dye Fura-2, respectively.

**Results:**

Acute exposure of PC12 cells to 6-OH-BDE-47 (5 μM) induced vesicular catecholamine release. Catecholamine release coincided with a transient increase in [Ca^2+^]*_i_*, which was observed shortly after the onset of exposure to 6-OH-BDE-47 (120 μM). An additional late increase in [Ca^2+^]*_i_* was often observed at ≥1 μM 6-OH-BDE-47. The initial transient increase was absent in cells exposed to the parent compound BDE-47, whereas the late increase was observed only at 20 μM. Using the mitochondrial uncoupler carbonyl cyanide 4-(trifluoromethoxy)phenylhydrazone (FCCP) and thapsigargin to empty intracellular Ca^2+^ stores, we found that the initial increase originates from emptying of the endoplasmic reticulum and consequent influx of extracellular Ca^2+^, whereas the late increase originates primarily from mitochondria.

**Conclusion:**

The hydroxylated metabolite 6-OH-BDE-47 is more potent in disturbing Ca^2+^ homeostasis and neurotransmitter release than the parent compound BDE-47. The present findings indicate that bioactivation by oxidative metabolism adds considerably to the neurotoxic potential of PBDEs. Additionally, based on the observed mechanism of action, a cumulative neurotoxic effect of PBDEs and *ortho*-substituted polychlorinated biphenyls on [Ca^2+^]*_i_* cannot be ruled out.

Increasing concentrations of brominated flame retardants, in particular polybrominated diphenyl ethers (PBDEs), in the environment, human food chain, and human tissues raise concern about possible neurotoxic effects. High concentrations of PBDEs and the structurally related polychlorinated biphenyls (PCBs) increase intracellular Ca^2+^ concentrations ([Ca^2+^]*_i_*) in cultured neuronal cells, likely through mobilizing Ca^2+^ from intracellular stores (for review, see Mariussen and Fonnum 2006). Such a xenobiotic-induced increase in [Ca^2+^]*_i_* is of particular concern because this increase, in addition to being essential for multiple physiologic and pathologic processes, is the trigger for vesicular release of neurotransmitters (exocytosis). This correlation between increased [Ca^2+^]*_i_* and the occurrence of exocytosis has been widely studied in neurons (for review, see Barclay et al. 2005) and neuroendocrine cells, including rat pheochromocytoma PC12 cells (for review, see Garcia et al. 2006). Evidence of oxidative metabolism of PBDEs is accumulating, but the neurotoxic potential of hydroxylated PBDE metabolites and their ability to affect Ca^2+^ homeostasis is still unknown.

In most biotic samples, 2,2′,4,4′-tetra-bromodiphenyl ether (BDE-47) is the predominant PBDE congener (Hites et al. 2004). Neonatal exposure to this PBDE congener induces neurobehavioral changes (Eriksson et al. 2001b) and reduces long-term potentiation (LTP) in mouse hippocampal slices (Dingemans et al. 2007). Analysis of brain tissue from BDE-47–exposed mice revealed that alterations in the composition of postsynaptic density proteins and kinase activity might play a role in the reduction of synaptic plasticity (Dingemans et al. 2007). The doses of BDE-47 resulting in impaired learning and memory and reduced LTP measured in hippocampal slices were estimated (using a distribution study; Staskal et al. 2006a) to result in peak brain concentrations of approximately 1 μM, whereas acute toxic effects of BDE-47 were seen *in vitro* only at concentrations ranging from 3 to 20 μM (Coburn et al. 2008; Dingemans et al. 2007.

The results of *in vitro* endocrine studies (focusing mostly on 6-hydroxy-2,2′,4,4′-tetrabromodiphenyl ether; 6-OH-BDE-47) on interactions with the estrogen and thyroid hormone receptor systems indicate that hydroxylated metabolites of PBDEs are more potent than the parent compounds (Cantón et al. 2005, 2006; Harju et al. 2007; Meerts et al. 2001). The conversion of PBDEs to hydroxylated metabolites was confirmed by recent toxicokinetics studies (Huwe et al. 2006; Malmberg et al. 2005; Marsh et al. 2005; Staskal et al. 2006b). Additionally, marine sponges can produce *ortho*-OH-PBDEs (Hakk and Letcher 2003). Hydroxylated metabolites have been detected in blood from wildlife and humans (for review, see Hakk and Letcher 2003). Therefore, we investigated the effects of 6-OH-BDE-47, a hydroxylated metabolite of the environmentally relevant PBDE congener BDE-47, on Ca^2+^ homeostasis and vesicular catecholamine release in PC12 cells to compare its neurotoxic potential with that of the parent compound.

## Methods

### Chemicals

BDE-47 and 6-OH-BDE-47 were synthesized and purified (~ 99% purity) at the Wallenberg Laboratory of Stockholm University as described by Marsh et al. (1999). Dibenzo-*p*-dioxins and dibenzofurans were removed from the PBDEs with a charcoal column as described by Örn et al. (1996). All other chemicals, unless otherwise stated, were obtained from Sigma-Aldrich (Zwijndrecht, the Netherlands).

### Cell culture

Rat pheochromocytoma (PC12) cells (Greene and Tischler 1976) obtained from the ATCC (American Type Culture Collection, Manassas, VA, USA) were cultured for up to 15 passages in RPMI 1640 medium (Invitrogen, Breda, the Netherlands) supplemented with 5% fetal calf serum and 10% horse serum (ICN Biomedicals, Zoetermeer, the Netherlands). For Ca^2+^ imaging experiments, we subcultured undifferentiated PC12 cells in poly-l-lysine–coated glass-bottom dishes (MatTek, Ashland, MA, USA) as described previously (Dingemans et al. 2007). For amperometric recordings, the cells were differentiated for 3–5 days with 5 μM dexamethasone to enhance exocytosis, as described previously by Westerink and Vijverberg (2002).

### Cell viability assay

We used cell density as an indicator of cell viability. After 20 min of exposure to dimethylsulfoxide (DMSO) or 20 μM 6-OH-BDE-47, cells were cultured in fresh cell culture medium for another 24 hr. After replacing the culture medium, which washes away most dead, detached cells, and trypan blue inclusion, which stains the remaining dead cells, we determined the proportion of the surface of the cell culture dish occupied by living PC12 cells in triplicate for three dishes per experimental condition.

### Amperometry

Amperometric recordings of K^+^-evoked and spontaneous vesicular catecholamine release from dexamethasone-differentiated PC12 cells using carbon fiber microelectrodes were made as described previously (Dingemans et al. 2007; Westerink and Vijverberg 2002). Following 1 min of baseline recording, we superfused PC12 cells for 15 sec with high K^+^-containing saline (K^+^ increased to 125 mM and Na^+^ lowered to 5.5 mM) to determine their responsiveness. Cells were allowed to recover for 2 min before a 15-min exposure to BDE-47 or 6-OH-BDE-47 to investigate acute effects on vesicular catecholamine release. Recordings were performed at room temperature. To ensure exclusion of nonresponsive or extraordinary cells, we determined basal release frequency for 22 cells. Cells that showed a basal release frequency larger than the average + 2 standard deviations were considered to have an extraordinary high release frequency. Based on these findings, we excluded cells with a basal release frequency > 5/min. Similarly, cells with an evoked release frequency < 16/min were excluded. We used the resulting 20 cells for further data analysis.

### Intracellular Ca^2+^ imaging

We measured changes in [Ca^2+^]*_i_* using the Ca^2+^-responsive fluorescent ratio dye Fura-2 as described previously (Dingemans et al. 2007). Briefly, cells were loaded with 5 μM Fura-2 AM (Molecular Probes; Invitrogen) in external saline (containing 1.8 mM CaCl_2_, 24 mM glucose, 10 mM HEPES, 5.5 mM KCl, 0.8 mM MgCl_2_, 125 mM NaCl, and 36.5 mM sucrose, adjusted to pH 7.3 with NaOH) for 20 min at room temperature; this was followed by 15 min de-esterification in external saline. The cells were then placed on the stage of an Axiovert 35M inverted microscope (Zeiss, Göttingen, Germany) equipped with a TILL Photonics Polychrome IV (TILL Photonics GmBH, Gräfelfing, Germany). Fluorescence evoked by 340 and 380 nm excitation wavelengths (F340 and F380) was recorded every 12 sec at 510 nm with an Image SensiCam digital camera (TILL Photonics GmBH). The digital camera and polychromator were controlled by imaging software (TILLvisION, version 4.01), which was also used for data collection and processing. We further analyzed changes in the F340/F380 ratio, reflecting changes in [Ca^2+^]*_i_*, using custom-made Excel macros (Microsoft Corp., Redmond, WA, USA). After 5 min baseline recording, cells were exposed to 0.2–20 μM BDE-47 or 6-OH-BDE-47. Maximum and minimum ratios were determined after 25 min recording (20 min exposure) by addition of ionomycin (5 μM) and ethylenediamine tetraacetic acid (EDTA; 17 mM) as a control for experimental conditions.

Where applicable, cells were washed with Ca^2+^-free external saline (containing 10 μM EDTA to remove residual extracellular Ca^2+^) just before the imaging experiments. In specific experiments, intracellular Ca^2+^ stores were emptied by incubation with 1 μM thapsigargin (TG) and 1 μM carbonyl cyanide 4-(trifluoromethoxy)phenylhydrazone (FCCP) in Ca^2+^-free external saline for 10 min. FCCP depolarizes the mitochondrial membranes, resulting in the uncoupling of oxidative phosphorylation and subsequent Ca^2+^ release from mitochondria (Taylor et al. 2000). TG is a high-affinity inhibitor of sarcoplasmic/endoplasmic reticulum (ER) Ca^2+^ ATPase (SERCA). These compounds are commonly used under experimental conditions to empty intracellular Ca^2+^ stores (Toyoshima and Inesi 2004). To further distinguish between direct effects on ER and Ca^2+^ influx pathways, we used dantrolene as an inhibitor of Ca^2+^ release from the ER.

### Data analysis and statistics

To determine effects on [Ca^2+^]*_i_*, we used the normalized F340/F380 ratio. Any change in the normalized ratios to ≥ 1.1 was considered an increase and was used for further data analysis. We refer to a transient increase in [Ca^2+^]*_i_* reaching its peak value (amplitude) between 0 and 4.5 min after application as an initial increase. We consider an additional increase after cessation of the initial transient increase to be a late increase. In a number of experiments (4/33), the initial fast transient was absent, and instead, a slower transient increase was observed. Because it is unclear whether this was a delayed initial transient increase or a transient form of the late increase, we excluded these experiments from further analysis. All data are presented as mean ± SE from the number of cells indicated. Statistical analyses were performed using SPSS 12.0.1 (SPSS, Chicago, IL, USA). Categorical data were compared using Fisher’s exact and chi-square tests. We compared continuous data using Student’s *t*-test, paired or unpaired where applicable. Analysis of variance (ANOVA) and post hoc *t*-tests (corrected for multiple comparisons) were performed to investigate possible dose–response relationships. A *p*-value < 0.05 was considered statistically significant.

## Results

### 6-OH-BDE-47 increases catecholamine release in PC12 cells

Exposure of PC12 cells to a high concentration (20 μM) of the brominated flame retardant BDE-47 was previously shown to induce vesicular catecholamine release, coinciding with a gradual increase in [Ca^2+^]*_i_* (Dingemans et al. 2007). To investigate whether oxidative metabolism changes the ability of PBDEs to affect vesicular catecholamine release, we measured the effects of 6-OH-BDE-47, a hydroxylated metabolite of BDE-47. Although cytotoxicity has been reported after subchronic exposure (24 hr) to 2.5 μM 6-OH-BDE-47 (Cantón et al. 2005), 20 min of exposure to 20 μM 6-OH-BDE-47 did not have any effects on cell viability determined 24 hr later, suggesting the absence of acute cell toxicity (data not shown).

**Figure 1:**
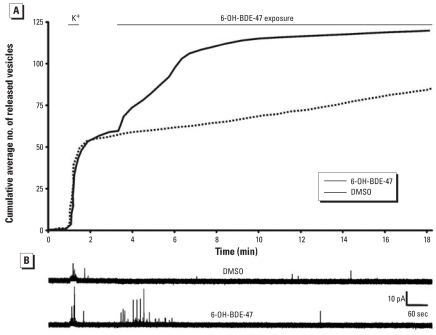
Catecholamine release in PC12 cells exposed to DMSO (*n* = 9) or 5 μM 6-OH-BDE-47 (*n* = 11) shown as the cumulative average number of released vesicles (*A*). Results clearly demonstrate that 6-OH-BDE-47 induced exocytosis. (*B*) Representative amperometric traces recorded from cells exposed to DMSO or 5 μM 6-OH-BDE-47.

To investigate whether exposure to 6-OH-BDE-47 has functional consequences for neuronal communication, we measured vesicular catecholamine release (Figure 1). First, cells were challenged for 15 sec with high K^+^-containing saline to determine their responsiveness. Responsive cells displayed depolarization-evoked release (at least 16 released vesicles/min), after which the release frequency returned to baseline values. During the first 2.5 min of a subsequent exposure to 5 μM 6-OH-BDE-47 (*n* = 9), the release frequency was enhanced from 1.0 ± 0.3 to 13 ± 5.3 events/min (*p* < 0.05). This enhancement did not occur in cells exposed to DMSO only (from 1.8 ± 0.5 to 1.7 ± 0.7 events/min, not significant; *n* = 11). After the burst of exocytotic activity, the release frequency in 6-OH-BDE-47–exposed cells declined to a value not significantly different from basal release.

### 6-OH-BDE-47 causes a biphasic increase ***in [Ca2+]*i** ***in PC12 cells.***

**Figure 2:**
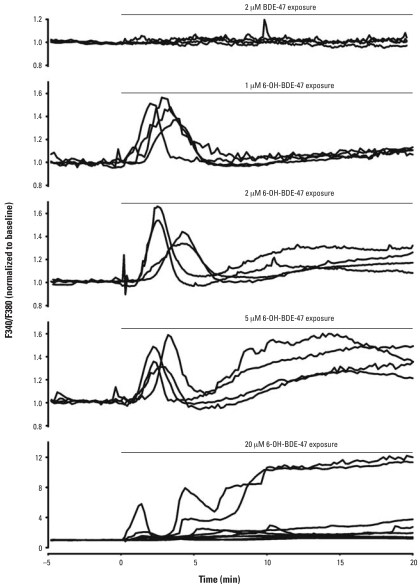
Biphasic increase in [Ca^2+^]*_i_* in PC12 cells after exposure to 6-OH-BDE-47. Results are shown as representative traces of normalized F340/F380 (reflecting [Ca^2+^]*_i_*) from individual PC12 cells exposed to 2 μM BDE-47 and 1, 2, 5, and 20 μM 6-OH-BDE-47 for 20 min, applied at *t* = 0, as indicated. Note the difference in scaling for 2 μM BDE-47 and 20 μM 6-OH-BDE-47.

**Figure 3:**
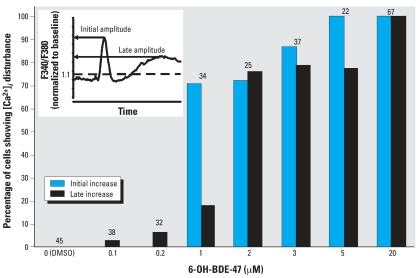
Concentration dependence of the occurrence of different types of [Ca^2+^]*_i_* disturbances during exposure to 6-OH-BDE-47. Bars indicate the percentage of cells showing an initial transient increase in [Ca^2+^]*_i_* or those showing a late increase in [Ca^2+^]*_i_*. For both processes, the percentage of cells displaying an increase is significantly higher than in control at concentrations ≥ 1 μM (initial: *p* < 0.001; late: *p* < 0.01). Data are shown from three to eight experiments per concentration; numbers above each bar indicate the number of cells used for data analysis. Inset: representative recording with the characteristics of the increase in [Ca^2+^]*_i_* used in this article (i.e., amplitude of the initial and late increase in [Ca^2+^]*_i_*).

To investigate whether the observed changes in neurotransmitter release are caused by a disruption of calcium homeostasis, we measured the effects of 6-OH-BDE-47 on the [Ca^2+^]*_i_*. Exposure of PC12 cells to 6-OH-BDE-47 (≥ 1 μM) resulted in a dose-dependent increase in [Ca^2+^]*_i_* in PC12 cells (Figures 2 and 3), whereas exposure to similar concentrations of the parent compound had no effects on [Ca^2+^]*_i_* (Figure 2) (Dingemans et al. 2007). The parent compound BDE-47 caused a gradual increase of [Ca^2+^]*_i_* only at 20 μM (data not shown; Dingemans et al. 2007), whereas exposure to 1 μM of the hydroxylated metabolite resulted in an initial transient increase in [Ca^2+^]*_i_* (Figures 2 and 3). At concentrations ≥ 1 μM, 6-OH-BDE-47 also caused an additional late increase in [Ca^2+^]*_i_* (Figures 2 and 3). The relative occurrences (percentages of cells showing an effect) of initial transient and late increases in [Ca^2+^]*_i_* increased with increasing concentrations of 6-OH-BDE-47 (Figure 3). Exposure to vehicle or 0.1 or 0.2 μM 6-OH-BDE-47 had no significant effect on [Ca^2+^]*_i_* (Figure 3).

**Figure 4:**
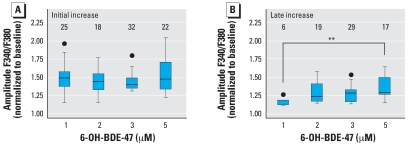
Amplitude of the initial and late increase in [Ca^2+^]*_i_* after exposure to 1–5 μM 6-OH-BDE-47 shown as boxplots of amplitudes reached during the initial transient (*A*) and late increase (*B*). Upper and lower borders of the box represent upper and lower quartiles; the line within the box is the median; whiskers represent lowest and highest values, and circles represent outliers. ANOVA analysis indicates no differences in the amplitude of the initial increase at different concentrations (*p* > 0.05). ANOVA analysis and post-hoc *t*-tests indicate that the amplitude of the late increase in [Ca^2+^]*_i_* increases with increasing concentration (*p* < 0.01). Data shown are from three to eight experiments per concentration; numbers above each box indicate the number of cells used for data analysis.

Exposure to 20 μM 6-OH-BDE-47 caused a large initial transient increase in [Ca^2+^]*_i_* (1.7 ± 0.1), and an even larger late increase (3.2 ± 0.4) compared to the normalized baseline. At this high concentration, non-specific effects are likely to occur. Therefore, we investigated the concentration dependence of the amplitude of the two types of increases in [Ca^2+^]*_i_* within the range of 1 μM (the lowest concentration where the effects occur) to 5 μM. ANOVA analysis indicated no relationship between the applied 6-OH-BDE-47 concentration and the amplitude of the initial Ca^2+^ transient (Figure 4A). Analysis of the late increase indicated that the amplitude of this increase is concentration dependent (Figure 4B), although the biologic relevance of this small change remains to be determined. The distinct temporal aspects combined with this observation on the concentration dependence suggest that distinct mechanisms underlie both phases of increasing [Ca^2+^]*_i_*.

### 6-OH-BDE-47–induced increase in [Ca^2+^]_i_ mainly originates from intracellular stores

**Figure 5:**
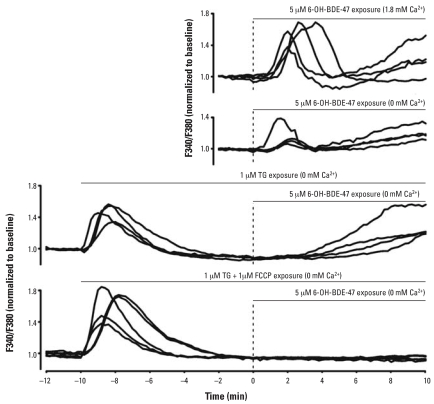
Release from intracellular Ca^2+^ stores in PC12 cells after exposure to 6-OH-BDE-47. Results are shown as representative traces of [Ca^2+^]*_i_* measurements of individual PC12 cells exposed to 5 μM 6-OH- BDE-47 (applied at *t* = 0; dashed line) in external saline (containing 1.8 mM Ca^2+^) and under Ca^2+^-free conditions as indicated for each panel. The initial transient increase is smaller under Ca^2+^-free conditions (note the different scale). When cells were pretreated with TG or TG + FCCP, an immediate increase in [Ca^2+^]*_i_*, corresponding to the emptying of intracellular stores, can be seen. Upon subsequent exposure to 6-OH-BDE-47, the initial transient increase, as observed under control conditions, is absent. Only in cells pretreated with TG and FCCP is the 6-OH-BDE-47–induced late increase also absent.

**Figure 6:**
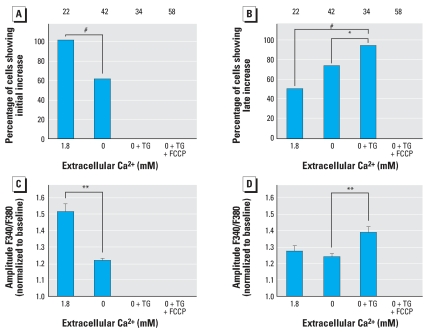
Occurrence and amplitudes of initial and late 6-OH-BDE-47–induced increases at different experimental conditions. The effects of 5 μM 6-OH-BDE-47 on [Ca^2+^]*_i_* were measured in external saline (1.8 mM Ca^2+^), Ca^2+^-free saline (0 mM Ca^2+^), Ca^2+^-free saline after pretreatment with TG (0 mM Ca^2+^ + TG), and Ca^2+^-free saline after pretreatment with both TG and FCCP (0 mM Ca^2+^ + TG + FCCP). Occurrence (*A*) and amplitude (*C*) of the initial increase in [Ca^2+^]*^i^*. Occurrence (*B*) and amplitude (*D*) of the late increase in [Ca^2+^]*_i_*. Data are from four experiments per treatment. Numbers above bars indicate the number of cells used for data analysis; values shown are mean ± SE for the number of cells indicated. *p < 0.05; **p < 0.01; ^#^*p* < 0.001.

To investigate the mechanisms underlying the observed increase in [Ca^2+^]*_i_*, we performed Ca^2+^ imaging experiments under Ca^2+^-free conditions to reveal whether extracellular Ca^2+^ is required. Both the initial transient and the additional late increase in [Ca^2+^]*_i_* were still present under Ca^2+^-free conditions. However, the occurrence and amplitude of the initial increase were significantly higher under physiologic Ca^2+^ conditions (1.8 mM; Figures 5 and 6). The occurrence and amplitude of late increases were not altered under Ca^2+^-free conditions (Figures 5 and 6). From these data, we conclude that the initial increase depends only partially on extracellular Ca^2+^, whereas the late increase is independent of external Ca^2+^, indicating that the 6-OH-BDE-47–induced increase in [Ca^2+^]*_i_* largely relies on the release of Ca^2+^ from intracellular stores.

To identify the intracellular stores responsible for the observed increase in [Ca^2+^]*_i_*, we performed additional Ca^2+^ imaging experiments using PC12 cells in which mitochondrial and TG-sensitive intracellular Ca^2+^ stores were depleted by pretreatment with FCCP and TG, respectively. After depletion of ER Ca^2+^ stores with TG under Ca^2+^-free conditions, 5 μM 6-OH-BDE-47 was no longer able to evoke the initial transient increase in [Ca^2+^]*_i_*, but the late increase was still present (Figure 5). Both the occurrence and the amplitude of the late increase were larger after TG pretreatment compared with normal and Ca^2+^-free conditions (Figure 6), indicating a tight coupling between intracellular Ca^2+^ stores. After depletion of both mitochondrial and ER Ca^2+^ stores with FCCP and TG under Ca^2+^-free conditions, both the initial transient and the late increase in [Ca^2+^]*_i_* were completely absent. These combined data indicate that the initial transient increase in [Ca^2+^]*_i_*, which depends only partly on Ca^2+^ influx, is mainly caused by intracellular Ca^2+^ release from the ER, whereas the late increase is mainly due to Ca^2+^ release from mitochondria.

To investigate whether the influx of extra-cellular Ca^2+^ in the initial transient increase was related to emptying of the ER, we exposed dantrolene (100 μM)-pretreated PC12 cells to 5 μM 6-OH-BDE-47 under physiologic Ca^2+^ conditions. The occurrence and amplitude of the initial transient increase were markedly reduced under these conditions (data not shown), suggesting that store-operated Ca^2+^ entry (SOCE) largely accounts for the influx of extracellular Ca^2+^.

## Discussion

The results of the present study demonstrate that both the abundant PBDE congener BDE-47 and its hydroxylated metabolite 6-OH-BDE-47 increase [Ca^2+^]*_i_* in PC12 cells, although the hydroxylated metabolite does so at much lower concentrations. The initial transient and the late increase in [Ca^2+^]*_i_* are due to release of Ca^2+^ from endoplasmic and mitochondrial Ca^2+^ stores, respectively; extracellular Ca^2+^ also plays a role in the observed initial increase in [Ca^2+^]*_i_* during exposure to 6-OH-BDE-47. Interestingly, the initial increase in [Ca^2+^]*_i_* is temporally linked with vesicular catecholamine release, raising concern about effects of BDE exposure on neurotransmission.

The increase in [Ca^2+^]*_i_* is mainly caused by release of Ca^2+^ from intracellular stores, which are involved in controlling intracellular Ca^2+^ homeostasis and neurotransmitter release (for review, see Garcia et al. 2006). It is noteworthy that disruption of intracellular Ca^2+^ homeostasis by release of Ca^2+^ from intracellular stores and influx of extracellular Ca^2+^ is also considered an important factor in the neurotoxicity of PCBs (for review, see Fonnum et al. 2006; Kodavanti 2005).

The effects of 6-OH-BDE-47 on exocytosis and [Ca^2+^]*_i_* have been investigated in PC12 cells, which are widely used as an *in vitro* neuroendocrine model to study neurotransmitter secretion (for review, see Westerink and Ewing 2008). Possible origins for the 6-OH-BDE-47–induced increase in [Ca^2+^]*_i_* are influx of extracellular Ca^2+^ or release from intracellular Ca^2+^ stores. In adrenal chromaffin and PC12 cells, intracellular Ca^2+^ stores are ER, mitochondria, nucleus, and secretory vesicles. Influx via voltage-gated Ca^2+^ channels and SOCE channels, and efflux from the ER and the mitochondria, are tightly coupled and locally control the [Ca^2+^]*_i_* that regulates exocytosis (for review, see Garcia et al. 2006; Parekh and Putney 2005). The increase in [Ca^2+^]*_i_* following exposure to 6-OH-BDE-47 is also associated with an increase in vesicular catecholamine release in PC12 cells. The increase in catecholamine release was most apparent during the first 2.5 min of exposure, whereas release frequencies were no longer different between control cells and cells exposed to 6-OH-BDE-47 after 5 min of exposure (Figure 1). The strong temporal link between the 6-OH-BDE-47–induced initial transient increase in [Ca^2+^]*_i_* (by emptying of the ER and subsequent SOCE) and the 6-OH-BDE-47–induced burst of exocytotic activity strongly suggests a causal relationship. Because the observed late increase in [Ca^2+^]*_i_* (by Ca^2+^ release from mitochondria) has a smaller effect on [Ca^2+^]*_i_* than the emptying of the ER and subsequent SOCE (Figure 4) at concentrations <5 μM, the association between this late increase in [Ca^2+^]*_i_* and neurotransmitter release is likely to be of less toxicologic concern.

Because the initial peak is completely absent in TG-treated cells, we concluded that this increase in [Ca^2+^]*_i_* primarily originates from the ER. Another brominated flame retardant (tetrabromobisphenol A) has recently been shown to be a potent inhibitor of the SERCA Ca^2+^ pump (Ogunbayo and Michelangeli 2007). The reduced amplitude of the initial transient [Ca^2+^]*_i_* increase under Ca^2+^-free conditions (without TG pre-treatment) indicates that both an intracellular and extracellular Ca^2+^ component contribute to this transient increase. The extracellular Ca^2+^ component could be caused by a direct effect of 6-OH-BDE-47. However, the TG experiments suggest it is more likely that SOCE, through SOCE channels, in response to 6-OH-BDE-47–induced emptying of the ER, accounts for the involvement of extra-cellular Ca^2+^. SOCE is commonly observed in PC12 cells after depletion of Ca^2+^ stores (Bennett et al. 1998; Taylor and Peers 1999). When Ca^2+^ release from the ER is inhibited by dantrolene during exposure to 6-OH-BDE-47, the initial transient increase is markedly reduced, suggesting that a large part of the extracellular component is indeed an indirect effect of 6-OH-BDE-47 associated with SOCE. However, as a small initial transient increase can still be observed, it is not possible at present to exclude a direct effect of 6-OH-BDE-47 on other Ca^2+^ influx pathways.

In cells treated with TG and the mitochondrial uncoupler FCCP, both the initial and the late increase no longer occur after application of 6-OH-BDE-47. As the initial increase was already abolished by TG, these results indicate that the late increase in [Ca^2+^]*_i_* mainly originates from mitochondria. In TG-treated cells, the amplitude of the late Ca^2+^ increase is larger (Figure 6). A possible explanation for this enhancement of the late Ca^2+^ increase is the tight coupling between Ca^2+^ influx via voltage-gated and SOCE channels and efflux from intracellular stores (for review, see Garcia et al. 2006; Parekh and Putney 2005). This coupling predicts that mitochondria take up Ca^2+^ from the emptied TG-sensitive stores and will thus be filled to a larger extent. As a consequence, emptying of mitochondrial stores by 6-OH-BDE-47 will result in an enhancement of the late Ca^2+^ increase (Figure 6).

The present findings add to previous studies demonstrating that *ortho*-substituted (non-planar) PCBs increase [Ca^2+^]*_i_* in cultured neural cells and brain preparations (Howard et al. 2003; Inglefield and Shafer 2000; Kang et al. 2004; Kodavanti et al. 1993; Magi et al. 2005; Voie and Fonnum 1998; Wong et al. 1997). Inhibition of endoplasmic and mitochondrial Ca^2+^-ATPases, mobilization of Ca^2+^ from the ER through interaction with the inositol triphosphate (IP_3_)- and ryanodine receptors, and disruption of plasma, mitochondrial, and endoplasmic membranes have all been proposed as possible mechanisms. Furthermore, the commercial PBDE mixture DE-71, which contains 31.8% BDE-47, has also been shown to disrupt microsomal Ca^2+^ homeostasis (Kodavanti and Ward 2005). More recently, a gradual increase in [Ca^2+^]*_i_* in PC12 cells has also been reported for the environmentally relevant BDE-47, although only at 20 μM (Dingemans et al. 2007). Also, BDE-47, as well as 2,2′4,4′5-pentabromo-diphenyl ether (BDE-99), result in a reduced net Ca^2+^ uptake by microsomes and mitochondria isolated from frontal cortex, cerebellum, hippocampus, and hypothalamus of adult male rats measured after 20 min exposure to 3–30 μM (Coburn et al. 2008).

The underlying mechanisms of the (hydroxylated) PBDE-induced disruption of Ca^2+^ homeostasis should be investigated in more detail. Increasing evidence suggests that formation of reactive oxygen species (ROS) could be responsible for the observed effects on [Ca^2+^]*_i_*. In rat hippocampal neurons as well as in human SH-SY5Y cells and neutrophil granulocytes, BDE-47 has been shown to induce ROS formation at exposure concentrations of 41, 4, and 6 μM, respectively (He et al. 2008a, 2008b; Reistad and Mariussen 2005). However, the effect of hydroxylated BDE-47 on ROS formation remains to be determined. Mechanisms usually associated with increased ROS formation include activation of tyrosine kinase, IP_3_-kinase, protein kinase C, phospholipase C, and nicotinamide adenine dinucleotide phosphate (NADPH) oxidase; release of arachidonic acid; and disturbed and increased [Ca^2+^]*_i_* (Kodavanti and Derr-Yellin 2002; Kodavanti and Ward 2005; Reistad and Mariussen 2005). Because of the ether group connecting the phenyl rings in PBDEs, these compounds display a structural resemblance with *ortho*-substituted PCBs. It is therefore likely that disruption of Ca^2+^ homeostasis (for review, see Mariussen and Fonnum 2006), primarily caused by Ca^2+^ release from intracellular stores, is a common feature underlying the neurotoxicity of both *ortho*-PCBs and PBDEs.

Determination of the relative potency of 6-OH-BDE-47 and BDE-47 based on half maximal effective concentrations requires full dose–response curves with similar slopes and efficacy, which is not realistic due to the occurrence of nonspecific effects at high concentrations of 6-OH-BDE-47 and the relatively low potency of the parent compound and consequent solubility problems. Nonetheless, comparison of lowest observed effect concentrations clearly reveals that 6-OH-BDE-47 has a potency at least one order of magnitude higher than the parent compound BDE-47.

In the 1990s, an association between delayed human neurodevelopment and prenatal or neonatal exposure to PCBs was reported in cohort studies, which were corroborated by experiments demonstrating developmental neu-rotoxicity of PCBs (for review, see Winneke et al. 2002). Although epidemiologic evidence for a similar association of PBDEs is yet lacking, it has been established that exposure of mice to these environmental pollutants during brain development can cause toxic effects at doses much lower than those affecting adult brain function (Eriksson et al. 2001a). It is thus of particular concern that young children at critical stages of brain development are exposed to higher concentrations of PBDEs than adults. This high exposure is mainly associated with an increased exposure of children to house dust, which is an important source of PBDEs (Jones-Otazu et al. 2005). Additionally, global differences in PBDE body burden are observed, with average levels in North America being approximately 10 times higher than in Europe and Asia (Birnbaum and Cohen Hubal 2006). Very high serum concentrations of BDE-47 (as well as other PBDE congeners) have recently been measured in children working and living on a waste dumpsite in Nicaragua (Athanasiadou et al. 2008). These samples also have shown that hydroxylated PBDE metabolites bioaccumulate in human serum. The highest concentration of 6-OH-BDE-47 measured was 13 pmol/g lipid weight, corresponding to approximately 0.14 nM in blood (calculated using average physiologic values). These *in vivo* values are still orders of magnitude lower than those that exert effects in the present *in vitro* study. However, particular concern about neurotoxicity arises from the fact that comparable or even higher levels were observed for several other hydroxylated PBDE metabolites, for which even fewer toxicity data are available than for 6-OH-BDE-47.

In summary, exposure of PC12 cells to ≥1 μM 6-OH-BDE-47 increases exocytosis and [Ca^2+^]*_i_*, mainly via release from ER and mitochondria, whereas its parent compound BDE-47 causes comparable effects only at 20 μM. Furthermore, recent *in vivo* findings demonstrated that neonatal exposure of mice to BDE-47 causes permanent effects on neurobehavior (Eriksson et al. 2001b) and synaptic plasticity (Dingemans et al. 2007). Human exposure to hydroxylated PBDE metabolites results from uptake from natural sources and from internal oxidative metabolism of PBDEs (Hakk and Letcher 2003). In this respect, it should also be noted that exposure to PBDEs in children at the age of rapid brain development is disproportionally high (Jones-Otazu et al. 2005). The stronger Ca^2+^ homeostasis-disrupting effect of these hydroxylated metabolites is therefore a critical factor that should be taken into account in human PBDE risk assessment, in particular in relation to neurotoxicity and neurodevelopment. Based on the mechanism of action observed in the present study with PBDEs and those reported earlier for *ortho*-substituted PCBs (i.e., disruption of Ca^2+^ homeostasis), a cumulative neurotoxic effect (on [Ca^2+^]*_i_*) of both groups of compounds can not be ruled out. Further research should determine whether combined exposure to PBDEs and *ortho*-PCBs is of neurotoxicologic relevance in humans.
